# Anticoccidial activity of *Aloe Vera L*eafs’ aqueous extract and vaccination against *Eimeria tenella*: pathological study in broilers

**DOI:** 10.1007/s11259-023-10222-x

**Published:** 2023-09-22

**Authors:** Shahenaz M.H. Hassan, Rasha Zayeda, H. Elakany, Sohair Badr, A. Abou-Rawash, Hoda Abd-Ellatieff

**Affiliations:** 1 Alexandria Regional Laboratory, Animal Health Research Institute, Agriculture Research Center, Alexandria City, Egypt; 2 Animal Health Research Institute, Tanta Regional Laboratory, Tanta City, Egypt; 3 Department of Poultry and Fish diseases, Faculty of Veterinary Medicine, Damanhour University, Damanhour City, Egypt; 4 Pathology Department, Animal Health Research Institute Agriculture Research Center, Cairo City, Egypt; 5 Department of Pathology, Faculty of Veterinary Medicine, Damanhour University, Damanhour Cty, 25511 Egypt

**Keywords:** Anticoccidial activity of Aloe Vera, Anticoccidial vaccination, Anticoccidial activity of Aloe Vera and vaccination, Pathology of E. tenella in broilers

## Abstract

This study aimed to assess the efficacy of an anticoccidial vaccine and the anticoccidial activity of Aloe vera in broiler chickens infected with Eimeria tenella (E. tenella). A total of 225 healthy, sexless, one-day-old broiler chicks (avian48) from a commercial broiler company were randomized into nine experimental groups of 25 chicks. The groups were as follows: Group 1 (control, vaccinated, non-infected), Group 2 (vaccinated and infected with 5 × 104 sporulated oocysts), Group 3 (vaccinated, infected with 5 × 104 sporulated oocysts, and treated with Aloe vera), Group 4 (infected with 5 × 104 sporulated oocysts and treated with Aloe vera), Group 5 (positive control, infected with 5 × 104 sporulated oocysts), Group 6 (challenged with 5 × 104 sporulated oocysts and then treated with amprolium), Group 7 (treated with amprolium), Group 8 (blank control negative group), and Group 9 (treated with Aloe vera gel).

Various parameters were evaluated, including clinical signs, growth performance, oocyst shedding, hematological and immunological parameters, and pathological lesion scoring. The results demonstrated that Aloe vera improved growth performance, reduced oocyst shedding, and decreased caecal lesion scores in E. Tenella-infected broiler chicks. The use of Aloe vera in combination with either amprolium or anticoccidial vaccines provided a potential solution to the issues of drug resistance and drug residues.

In conclusion, this study provides valuable insights regarding the control of coccidiosis in broilers. Supplementing the chicken diet with Aloe vera had beneficial effects on the pathogenicity and infectivity of E. tenella, making it a cost-effective alternative as an herbal extract with no adverse side effects for coccidiosis control. These findings suggest that Aloe vera can be considered a potential candidate for inclusion in broiler diets for effective coccidiosis control.

**Supplementary Information:**

The online version contains supplementary material available at 10.1007/s11259-023-10222-x.

## Research Highlights

- Efficacy of an anticoccidial vaccine, and Aloe vera extracts in broiler chickens infected with E. tenella.
- Supplementation of Aloe vera had improved growth performance, reduced oocyst shedding, and decreased caecal lesion scores in infected broiler chicks.
- The combination of Aloe vera and anticoccidial vaccines showed promise for addressing drug resistance and residues.
- The clinical signs, growth performance, oocyst shedding, haematological and immunological parameters, and pathological lesion scoring were all evaluated.
- Aloe vera is a safe and cost-effective alternative to herbal extracts for the control of coccidiosis in broilers.
- The findings suggest incorporating Aloe vera into broiler diets for effective coccidiosis control.

**Supplementary Information:**

The online version contains supplementary material available at 10.1007/s11259-023-10222-x.

## Introduction

Eimeria, a contagious protozoan disease, poses a significant challenge in both livestock and non-production species due to its severity and resistance to treatment (Blake et al. 2021). The disease is caused by Apicomplexa-classified obligate parasites belonging to the Apicomplexa phylum (Noack et al. 2019). Coccidiosis is a disease whose global prevalence causes significant economic losses, particularly for broiler chickens. The robust resistance of Eimeria oocysts, the parasite’s reproductive forms, to environmental stress makes its control and eradication particularly difficult. These oocysts frequently contaminate water, litter, and feed, especially under inadequate hygiene management and farm biosecurity conditions.

E. tenella, a specific Eimeria species, intricately follows a two-stage life cycle within the host. The exogenous stage initiates with sporogony, commencing with the excretion of unsporulated oocysts in feces, which then undergo sporulation and subsequent infection. The endogenous stage includes schizogony and gametogony, during which infected oocysts release sporozoites and infect chickens. These sporozoites invade intestinal epithelial cells, resulting in invasion of the host cell. Merozoites develop into male or female microgametes (Sharman et al. 2010). Eventually, the fusion of male and female gametes results in the formation of a zygote. These zygotes develop into infective oocysts in the gut, then expelled via feces (Kinnaird et al. 2004).

Significant coccidia colonization causes variable damage to the intestinal tract and caecum tissue, resulting in decreased growth rate, impaired feed conversion, and overall reduced chicken performance and productivity (Macdonald et al. 2017).

Modern poultry production necessitates effective coccidiosis management through judicious husbandry practices, vaccination, and chemoprophylaxis. However, the extensive use of anticoccidial drugs has catalyzed drug resistance development across various parasites (Blake et al. 2021). Consequently, regulatory and public pressures have prompted reducing or eliminating chemoprophylaxis use, as evidenced by certain regions such as the European Union (Blake et al. 2021).

Therefore, alternative strategies have emerged to address these challenges. These include live anticoccidial vaccines, immunomodulators, prebiotics, and natural herbs or extracts (Boulton et al. 2018; Hamzić et al. 2015). Anticoccidial drugs remain essential for coccidiosis control, but the emergence of drug resistance poses a formidable challenge for their administration. The administration of these anticoccidial drugs has resulted in the emergence of drug resistance, posing a formidable challenge (Abbas et al. 2011; Chapman 1999). As a result, there is a persistent demand for efficient and safe alternatives. In this context, Aloe vera, an established herbal remedy, presents an intriguing avenue for research.

Despite the progress of Egypt’s poultry industry, coccidiosis continues to be a significant issue that causes substantial economic losses. Therefore, alternative strategies for coccidiosis control, including the use of natural products and herbal medicines, have gained popularity for coccidiosis control (Hassan et al. 2008; Orengo et al. 2012). Aloe vera, a versatile succulent traditionally utilized in various cultures, is of particular interest. Its extensive bioactive compound composition and documented applications in medicine, cosmetics, and the management of poultry diseases make it a promising area of research (Ahlawat and Khatkar 2011; Christaki and Florou-Paneri 2010; Mwale et al. 2005).

This study endeavors to evaluate the anticoccidial efficacy of an aqueous Aloe vera gel extract (AG) either independently or in combination with the standard anticoccidial drug amprolium and/or vaccination in broiler chickens experimentally infected with E. tenella.

## Materials and methods

### The preparation of *Aloe-vera* gel (10%)

*Aloe vera* was kindly provided by a local botanical garden and was identified by the Medicinal Plant, Toxicology, and Forensic Medicine Department, Faculty of Veterinary Medicine, Damanhour University. Fresh Aloe leaves were collected to extract Aloe gel. The leaves were cut using a pocket knife, the latex of the leaf was removed, and the gel was collected in a beaker. A 10% (w/v) concentrated infusion was prepared by placing 100 g of fresh gel in a glass bottle and adding one liter of hot, boiled, distilled water. As described previously by (Durrani et al. 2008), the bottle was shaken for 5–7 min to ensure thorough mixing and was then stored for 6–8 h at room temperature prior to use. The Aloe extracts were stored in a refrigerator at 4 °C for six days before use to prevent oxidation. Aloe vera gel at a concentration of 10% (w/v) was administered at a dose of 15 ml/liter in drinking water from 1 day until the end of the experiment.

### Coccidial vaccine

Broiler chicks were vaccinated with Fortegra® (Coccivac®B-52), manufactured by Intervet- Egypt-MSD Animal Health Company. Experimental broilers were vaccinated with the commercial Coccivac®B-52 containing live non-attenuated oocysts (*E. acervulina, E. maxima, E. mivati, and E. tenella*) via eye drop method at three days old.

### Experimental drug

The anticoccidial drug Amprolium (Amproxine 20%, water-soluble powder, Pharma Sweed Company Ltd., Egypt) was administered for routine treatment of avian coccidiosis. At the age of 22 days, 48 h after oocyst infection, the chicks were administered amprolium-containing drinking water (1.25 g/liter) at the dosage recommended by the manufacturer for five consecutive days, which is the typical duration for chemotherapy for coccidiosis.

### Collection and preparation of eimeria oocysts

The oocyst of coccidian species (*Eimeria tenella*) used in this study was kindly provided by the Department of Poultry Diseases, Faculty of Veterinary Medicine, Damanhour University. The oocysts were isolated from the caeca of naturally infected chickens and stored in 2.5% potassium dichromate solution at 4 °C (Alnassan et al. 2013). The oocysts were extracted from the cecal region of chickens that were naturally harboring the infection. Field-obtained E. tenella isolates were then subjected to a series of procedures following the methodology outlined by Davies and colleagues (Davies et al. 1963). The procedures included cultivation, identification, purification, and propagation steps. Using the McMaster technique described by Lane (1984), the infectious dose of 50,000 sporulated oocysts per chick in 2 mL of phosphate-buffered saline was calculated.

Before infection, microscopical examination of feces from all unvaccinated groups confirmed the absence of coccidial infection. In the unvaccinated groups, no oocysts were identified. Oocysts were detected in the feces of the vaccinated group on the fifth day after vaccination when feces from this group were examined. Chicks were infected at the 20th day of age by oral inoculation with 50,000 sporulated oocysts of E. tenella per 2 mL normal saline for each chick through oral gauge dosing by long rubber flexible tube as described by (Hong et al. 2006).

### Experimental design

Two hundred and twenty-five (n = 225) healthy, one-day-old, unsexed broiler chicks (avian48) were purchased from Fat Hens Company, Tanta, Egypt. The chicks were housed in a clean, well-ventilated room, previously fumigated with formalin and potassium permanganate mixture. The birds were reared under standard good hygienic conditions in a suspended floor pen system (0.1 m2 / bird). The birds were categorized into nine groups (25 birds per group). At one day of age, chicks were obtained from a commercial hatchery and fed a well-balanced commercial diet of anticoccidial-free drugs or antibiotics until the end of the experiment.

The chicks were randomly divided into nine experimental groups: Group 1 (controls; Vaccinated with coccivac B via eye drop at 3 days old and not infected; n = 25). Group 2 (Vaccinated with coccivac B via eye drop at 3 days old and infected by 5 × 104 sporulated oocysts orally at 20 days old; n = 25). Group 3 (Vaccinated with coccivac B via eye drop at three days old, treated with *Aloe vera* gel, 15 ml/liter from 1 day old until the end of the experiment, and infected by 5 × 104 sporulated oocysts orally at 20 days old). Group 4 (treated with *Aloe vera* gel, 15 ml/liter from 1 day old until the end of the experiment and challenged by 5 × 104 sporulated oocysts orally at 20 days old; n = 25). Group 5 (challenged with 5 × 104 sporulated oocysts orally at 20 days old; n = 25). Group 6 (challenged with 5 × 104 sporulated oocysts orally at 20 days old, then treated with amprolium by a dose of 1.25 g/liter drinking water at 22 days old, n = 25). Group 7 (treated with amprolium by a dose of 1.25 g/liter drinking water at 22 days old, n = 25). Group 8 (non-treated, non-infected, blank control group, n = 25). Group 9 (treated with *Aloe vera* gel, 15 ml/liter from 1 day old until the end of the experiment and not infected, n = 25).

All experimental groups of chicks were vaccinated against Newcastle disease using the Hitchner B1 strain (Biovet Egypt) on the seventh day of life and the LaSota strain vaccine (Intervet Company) on the eighteenth day. In addition, birds were vaccinated against Infectious Bursal Disease at two weeks of age via drinking water (Gumboro, 228 Intervet Company) (Table 1).

**Table 1:** Experimental design including different treatments assigned to different groups

Treatment	Groups	Delivering strategy	Age of the broilers (days)
Vaccinated with coccivac B and not infected (control group)	G1	- Eye drop	- 3 days
Vaccinated with coccivac B, and challenged group.	G2	- Eye drop - Oral dose of 5 × 10^4^ sporulated oocysts in 200 µl / normal saline per chick	- 3 days - 20 days
Vaccinated with coccivac B, treated with *Aloe vera* gel and infected with *Eimeria tenella* group.	G3	- Eye drop - *Aloe vera* gel extract 15 ml*/*liter in drinking water - Oral dose of 5 × 10^4^ sporulated oocysts in 200 µl / normal saline per chick	- 3 days - From 1 day old till the end of experiment - 20 days
Treated with *Aloe vera* and infected with *Eimeria tenella* group.	G4	- *Aloe vera* gel extract 15 ml*/*liter in drinking water - Oral dose of 5 × 10^4^ sporulated oocysts in 200 µl / normal saline per chick	- From 1 day old till the end of the experiment - 20 days
Not- treated, infected with *Eimeria tenella* group.	G5	- Oral dose of 5 × 10^4^ sporulated oocysts in 200 µl / chick of normal saline	- 20 days
Infected with *Eimeria tenella* and then treated with amprolium.	G6	- Oral dose of 5 × 10^4^ sporulated oocysts in 200 µl / chick of normal saline - 1.25 g/liter drinking water	- 20 days - 22 days
Amprolium treated and not-infected control group.	G7	- 1.25 g/liter drinking water	- 22 days
Non- treated, non- infected (blank control group).	G8	----------	----------
Treated with *Aloe vera* and not infected group.	G9	- 15 ml*/*liter in drinking water	- From 1 day old till the end of the experiment

### Efficacy parameters

#### Performance parameters

All chicken groups were weighed individually in the morning at 1, 7, 14, 21, 28, and 35 days of age. Daily feed consumption and daily weight gain were recorded. Moreover, the mean weight gains and FCR for each group were also determined (Holdsworth et al. 2004). The live body weight was determined weekly by weighing all chicks of all groups. Weekly body weight gain was calculated by subtracting body weight between consecutive measurements.

***Feed Consumption (FC) and Feed Conversion Ratio (FCR).*** The feed consumption (FC) was calculated by dividing the amount of feed consumed in grams (by a specific group) during the week by the number of chicks of this group during the same week. The feed conversion ratio was determined by dividing the amount of feed consumed by a chick in grams per week by the chick’s weight gain in grams per week.

$${\rm{FCR}}\,{\rm{ = }}\,\frac{{{\rm{Feed}}\,{\rm{intake}}\,\left( {\rm{g}} \right)\,{\rm{bird/week}}}}{{{\rm{Body}}\,{\rm{weight}}\,{\rm{gain}}\,\left( {\rm{g}} \right)\,{\rm{bird/week}}}}$$

#### Mortality or survival rate

The number of dead birds was recorded throughout the experiment.

The mortality rate was estimated by the following equation:

$${\rm{Mortality}}\,{\rm{rate}}\,\left( {\rm{\% }} \right)\,{\rm{ = }}\,\frac{{{\rm{Number}}\,{\rm{of}}\,{\rm{deaths}}\,{\rm{in}}\,{\rm{a}}\,{\rm{specified}}\,{\rm{period}}\,{\rm{ \times }}\,{\rm{100}}}}{{{\rm{Total}}\,{\rm{population}}\,{\rm{during}}\,{\rm{that}}\,{\rm{period}}}}$$

#### Blood Collection and Hemato-Immunological analysis

Blood samples were collected from the wing veins of five birds from each experimental group. Two blood samples were collected, one with EDTA for hematological analysis and the other for separation serum for immunological analysis. The hematological parameters like red blood cell count (RBCs), Haemoglobin Concentration (HGB), and Total leukocytic count (WBCs) were determined using an automatic vet CBC counter (Sysmex XT 2000 iV Corporation, KOBE, Japan) in accordance with the manufacturer’s instructions. Serum was collected at 6 dpi for Nitric oxide (NO) evaluation and at 9 dpi to measure IFN-γ and IL-4 inflammatory cytokines levels using chicks specific enzyme-linked immunosorbent assays (ELISA) kits, according to the manufacturer’s instructions (R&D Systems Inc., Minneapolis, MN, USA).

#### Gross Caecal Lesion Scoring

Bloody diarrhea was recorded at 4th dpi until 8th dpi, and the score was rated from 0 (normal) to 4 (complete bloody diarrhea). Five birds per replicate were randomly selected and weighed for lesion scoring on day 7th post-infection until the end of the experiment. The cecum was removed and opened, and the lesion scoring was performed using the method described by (Raman et al. 2011). Based on the gross caecal lesions, such as bloody diarrhea, the thickness of intestinal walls, and petechiae, the lesion scores were ranked from 0 to 4, according to the severity of the caecal lesions as 0 represented the completely normal cecum with no lesions, scores 1, 2, 3 and 4 represented approximately 25%, 50%, 75%, and extremely panic severe lesions and death, respectively.

#### Parasitological examination (Oocysts count)

E. tenella oocyst /gram of caecal feces was measured using the McMaster method (Chand et al. 2016; Hodgson 1970). The fecal sample was freshly collected daily from 6 to 10 dpi from each group, and their average was calculated (Holdsworth et al. 2004). The actual number of oocysts was calculated using the following formula: calculated using the following formula:

$${\rm{Number}}\,{\rm{of}}\,{\rm{oocytes}}\,{\rm{per}}\,{\rm{gram}}\,{\rm{of}}\,{\rm{fecal}}\,{\rm{matter}}\,{\rm{ = }}\,\frac{n}{{{\rm{0}}{\rm{.1}}}}{\rm{ \times 100 \times 0}}{\rm{.1}}$$

Where:

n = number of oocysts counted,

0.10 = volume of the McMaster counting chamber,

100 = 100 ml of water that the litter is soaked in,

0.1 = correction for 10 g of litter originally taken.

### Histopathological examination

Caecal tissue (4 cm) was collected from 5 experimental birds of each group, gently rinsed with phosphate-buffered saline (PBS), and then preserved in 10% neutral buffered formalin for histopathological examination. After proper fixation, the tissue specimens were trimmed, washed in running tap water, dehydrated in different ascending grades of ethyl alcohol, cleared in xylene, and embedded in paraffin. The paraffin embedding blocks were sectioned at 5 μm thickness and stained with Haematoxylin and Eosin (H&E stain) (Bancroft and Gamble 2008) and the histopathological lesions scoring was assigned (Korver and Klasing 1997). Another score was given microscopically for the parasite density (percentage parasitized epithelium) per cross-section of the cecum on a scale of 0–4: 1 = > 0–25%, 2 = > 25–50%, 3 = > 50–75%, 4 = > 75–100%.

### Statistical analysis

The data were expressed as mean followed by (standard error), i.e., mean ± SE. One-way analysis of variance ANOVA was carried out to determine the variance of the data registered, followed by Turkey’s test to detect significant differences among the control and experimental groups. In all the analyses, the confidence level was held at 95%, and P ≤ 0.05 was set for significance. Statistical analysis was performed using the method cited in Petrie and Watson (1999), and the whole data was fed into Microsoft Excel 2010, a computerized program using SPSS Inc. (2011).

## Results

### Anticoccidial index

The results for the anticoccidial index, which includes daily body weight (BW), body weight gain (BWG) in grams, feed intake (FI), and feed conversion ratio (FCR), during pre, during, and post-E. tenella infection periods are presented in Supplementary Tables 1, 2 and 3. No significant differences were observed between experimental groups for BW, BWG, FI, or FCR during the pre-infection period. At the fifth week of age, the BW and BWG of non-infected and Aloe vera-treated chickens (G9) were significantly higher than the normal control group (G8). This was followed by the non-infected, amprolium-treated group (G7). The vaccinated non-infected group (G1) had lower BW and BWG, while G5, G3, G2, G4, and G6 had the lowest BW and BWG, respectively. In terms of FCR, the infected non-treated group (G5) had the lowest value at 28 and 35 days of age, while the Aloe vera non-infected group (G9) had the highest values. During infection, there were few statistically significant differences between the coccidial-vaccinated infected group (G2) and the vaccinated and Aloe vera-treated infected group (G3) and the amprolium-treated infected group (G6). However, G3 was numerically superior, indicating that the addition of Aloe vera to the coccidial vaccine improved FCR. G9 exhibited the highest FCR, while G5 exhibited the lowest. The results suggest that adding Aloe vera to broiler chicks’ drinking water may improve their growth performance and resistance to coccidiosis, as depicted in Table 4.

### Hematological parameters

Hematological parameters are shown in Supplementary Table 5. At 9 dpi, a significant increase in RBC count and Hgb concentration was observed in G9, followed by G7, G6, and G1, compared to the negative control group (G8). No significant differences were noted between G2, G3, and G4. The positive control group (G5) had the lowest RBCs and Hb concentration values among all groups. G9 had the highest values for these parameters, followed by G1 and G7. No significant differences were observed among G2, G3, G4, and G6. The highest total WBC count was documented in G5, followed by G4, G3, G2, G6, and G1, respectively. No significant differences were recorded between G9 and the negative control group (G8). However, the non-infected group treated with amprolium (G7) had the lowest total WBC count.

Regarding NO production, the infected group (G5) expressed the highest value, significantly different from the negative control group (G8). G6, G4, G2, G3, G1, and G9 followed in decreasing order of NO production, while G7 displayed the lowest value. In terms of IFN-γ production, G5 had the highest value, which was significantly different from G3. No significant differences were observed between G2 and G4 or between G6 and G7. G9 had a reduced value compared to all other groups, but this value was slightly higher than that of the negative control group (G8). With regards to IL-4 production, G5 had the highest value, followed by G1, while the lowest values were seen in G2, G6, and G4. On the other hand, G7, G3, and G9 had an increase in IL-4 value compared to the negative control group (G8), as depicted in Table 6.

### Clinical signs

The chickens in the positive control group (G5) exhibited several clinical symptoms following the coccidial challenge, including pronounced weakness, depression, emaciation, rough feathers, pallor, aversion to food, a propensity to seek out warm places, shaky movement, drooping wings, pale combs, and dehydration. On the fifth day post-exposure, the diarrhea progressed from being watery and white to bloody. Bloody diarrhea was observed in all coccidial-exposed groups but was less severe in G5 than in the other groups. None of the chickens in groups G1, G8, or G9 displayed watery or bloody diarrhea, and no changes in the consistency of their feces were observed. Nonetheless, chickens in groups G6, G4, G2, and G3 exhibited varying degrees of illness. The chickens in groups G6, G4, and G2 were clinically healthy, but they exhibited a decrease in feed intake and diarrhea that ranged from slight and white to bloody. Compared to the other groups, G3 chickens exhibited more significant weakness, wasting, and bloody diarrhea.

### Mortality rate

According to the study’s findings, no fatalities were observed in groups G1, G7, G8, and G9. 4th and 5th week mortality rates were 24%, 16%, 12%, 12%, and 8% in G5, G3, G4, G2, and G6 groups, respectively. Prior to the third week of age, no mortalities were recorded in any of the experimental groups (Table 7).

### Parasitological findings and oocyst shedding

The present study investigated the effect of a coccidial vaccine, an anticoccidial drug (amprolium), and Aloe vera extract on oocyst shedding (OPG) in chickens infected with E. tenella (Table 8.). The results demonstrated that treatment with amprolium significantly reduced the mean OPG values compared to the positive control group, with OPG decreasing from 35.800 to 3.000 and from 197.300 to 30.000 in G6 and G5, respectively, at 7 and 10 dpi. The highest OPG was observed on day seven post-implant, followed by a gradual decline on days eight, nine, and ten pi. Additionally, treatment with Aloe vera extract significantly decreased oocyst loss, with a gradual decrease observed between 7 and 10 days post-infection. The infected and Aloe vera-treated group (G4) was found to reduce oocyst shedding similarly to the anticoccidial drug (amprolium). The vaccinated infected group (G2) also demonstrated a significant decrease in OPG values, albeit not to the same extent as G6 or G4. Aloe vera combined with the vaccine (Group 3) had an unsatisfactory effect on oocyst shedding compared to Groups G6, G4, or G2, but overall, these results were deemed promising compared to the positive control group (G5).

### Pathological findings and lesion scoring

The post-mortem examination of the positive control group (G5) at 7–15 dpi revealed severe petechial hemorrhage in the serosa of the unopened caeca, distended caeca, bloody cores formation in the distal end of the pouch, which was contracted and shortened with hemorrhagic thickened corrugated mucosa and anemic pale liver. At 15 dpi, the caecal core became harder and stuffed by the accumulation of sloughed mucosal surface material, with blood forming that hard caecal core. These findings indicate severe coccidiosis-associated lesions in the positive control group.

No significant differences in caecal lesion scores were recorded between the infected amprolium-treated group (G6), infected Aloe vera-treated group (G4), vaccinated infected group (G2), and vaccinated infected Aloe vera-treated group (G3) at 7 and 15 dpi. However, these groups had significantly lower caecal lesion scores compared to the positive control group (G5). The negative control group (G8) demonstrated zero scores, indicating no pathological changes or coccidiosis-associated lesions. (Supplemntary Table 9)

### Histopathology

Histopathological examination results of the caeca of broiler chickens infected with E. tenella and treated with different methods (Aloe vera extract, amprolium, vaccination) revealed various degrees of lesions and inflammatory responses.

In the non-treated infected group (G5), the cecal villi exhibited desquamation of the mucosal epithelium, blunting, and degeneration. Several developmental stages of Eimeria, including gametocytes and oocysts, were identified, as well as a massive infiltration of heterophils and mononuclear cells in the lamina propria. There were merozoite-containing schizonts in the lamina propria of caecal crypts and submucosal glands. There were shed enterocytes, schizonts, free merozoites, and inflammatory cell infiltrations in the cecal lumen.

**Fig. 1:**
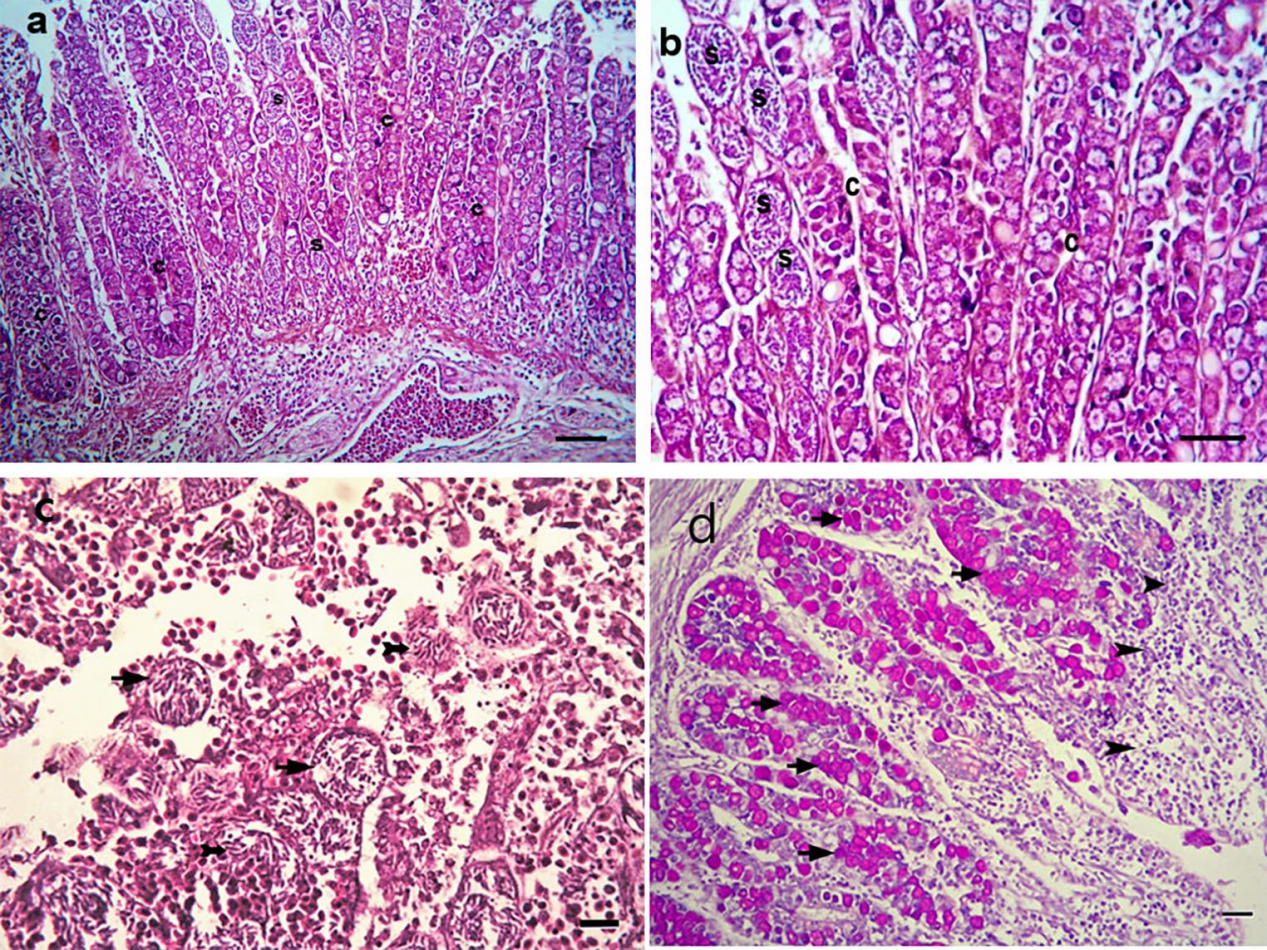
Cecum of chicken infected with Eimeria tenella and non-treated (**G5**) 7- days after infection (**a**) showing impaction of epithelium with myriads of various stages of Eimeria in mucosal crypts (s) with shedding of them into the lumen, with inflammatory cells infiltrations in the lamina propria and sub mucosa(*) H& E, bar = 40 µ ; (**b**) Higher magnification of part of villi notice sustitutionof of the degenerated and necrosed mucosal epithelium with myriads of the developing stages of Eimeria stages in mucosal crypts S = developing schizonts, c = leumen of degenerated crypts, H&E bar = 20 µ.; (**c**) degenerated mucosa, showing clusters of schizonts, (arrow) free banana shape merozoits (arrow head), free RBCS and inflammatory cells infiltration in the intestinal lumen H& E, bar = 10 µ.; (**d**), showing PAS positive Eimeria stages (oocysts) filling mucosal glandular, crypt epithelium PAS stain, bar = 20 µ

The histopathological examination of the cecum of chickens infected with E. tenella and non-treated (G5) at the seventh dpi revealed severe damage to the mucosal epithelium of the caecal villi, with desquamation and blunting of the villi. Various developmental stages of Eimeria (gametocytes, oocysts) affected the mucosal epithelium lining the villi and submucosal glands, leading to massive infiltration of heterophils and mononuclear cells in the lamina propria. Clusters of schizonts containing merozoites were constantly present in the lamina propria of caecal crypts, submucosal glands, and necrotic degenerated submucosal glands. Furthermore, sloughed enterocytes, schizonts, free merozoites, and inflammatory cell infiltrations were observed within the caecal lumen (Fig. 1).

**Fig. 2:**
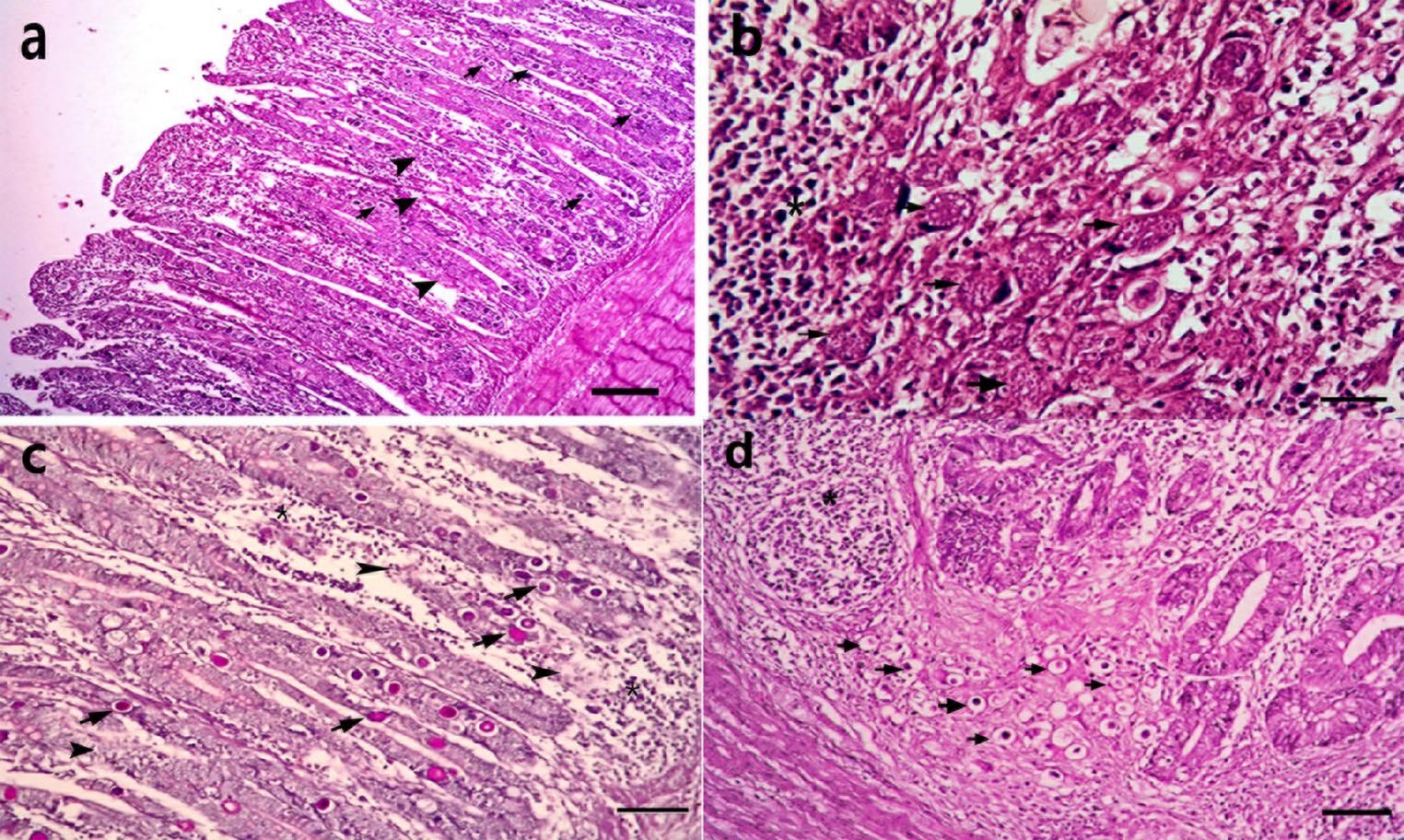
Cecum of chicken vaccinated before infection with *Eimeria tenella* (**G2**) 7- days after infection shpwing (**a**) blunting cecal villi, Heterophilic and mononuclear cells infiltrations in lamina propria and sub mucosa, and presence of developmental stages of eimeria (arrow) in some degenerated mucosal epithelium (arrow head) H& E, bar = 40 µ.; (**b**) Most of the schizonts (arrow) and other developing stages were degenerated in the necrosed sub mucosal glandular epithelium (arrow head), and surrounded by mononuclear cells infiltration (*)H& E, bar = 20 µ.; (**c**) few numbers of PAS positive oocysts (arrow) in degenersated epithelium (arrow head) PAS stain, bar = 20 µ.; (**d**) after 15th dpi revealed degenerated oocysts in lamina propria surrounded by mononuclear inflammatory cells (*) H& E, bar = 20 µ

**Fig. 3:**
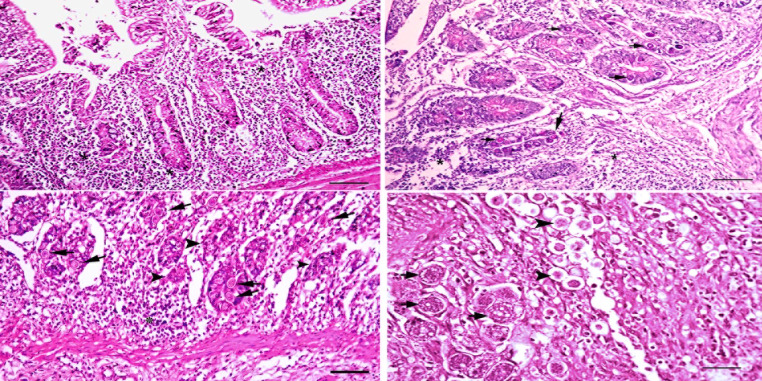
Cecum of chicken treated with amprolium and infected with Eimeria tenella (**G6**) after 7- days from infection (**a**) showing low number of Eimeria stages in necrotic and degenerated mucosal epithelium. With very heavy mononuclear cells infilterations (*) H& E, bar = 20 µ., (**b**) Cecum from the same group showing low number of Eimeria stages in the sub mucosal glandular epithelium (arrow), with heavy mononuclear cells infilterations (*) H&E stain, bar = 20 µ (**c**) Cecum from the same group after 15th dpi showing degenerated oocyst (arrow)in and necrotic mucosal glands (arrow head) H&E, bar = 20 µ. (**d**) Most of the schizonts (arrow) and other developing stages (arrow head) in degenerated and necrosed mucosal epithelium H& E, bar = 20 µ

The histopathological examination of the cecum of chickens vaccinated before infection with E. tenella (G2) at the seventh dpi revealed some degree of damage to the caecal villi, with blunting of the villi and infiltration of heterophilic and mononuclear cells in the lamina propria and submucosa. Developmental stages of Eimeria were detected in some degenerated mucosal epithelium, and most of the schizonts and other developing stages were degenerated in the necrosed submucosal glandular epithelium. At 15 days post-infection, a decrease in the number of oocysts was accompanied by mild desquamation of the caecal villi’s epithelial lining, hyperplasia of goblet cells, and marked inflammatory cell infiltrations. With PAS stain, very few developing stages were detected, indicating a decrease in the number of Eimeria developmental stages in the tissues (Fig. 2),

The histopathological examination of the cecum of chickens treated with amprolium and infected with E. tenella (G6) revealed marked cellular infiltration in the lamina propria and submucosa, with few numbers of Eimeria developmental stages detected in the degenerated epithelium. Most of the developmental stages in the submucosal glandular epithelium (schizonts and oocysts) were found to be degenerated. Fibrous connective tissue proliferation in the submucosa, extending in between the necrotic degenerated muscular layer, was also observed (Fig. 3).

At seven days post-infection, a histopathological examination of the cecum of chickens treated with Aloe vera gel extract and infected with E. tenella (G4) revealed mild desquamation of the epithelial lining of the caecal villi, with hyperplasia of epithelial goblet cells, a limited number of Eimeria developmental stages, mild inflammatory cell infiltrations, and submucosal edema. The submucosal glandular epithelium revealed the presence of a small number of Eimeria developmental stages, the majority of which were degenerated. The majority of submucosal glands were normal, with the disappearance of Eimeria oocysts.

**Fig. 4:**
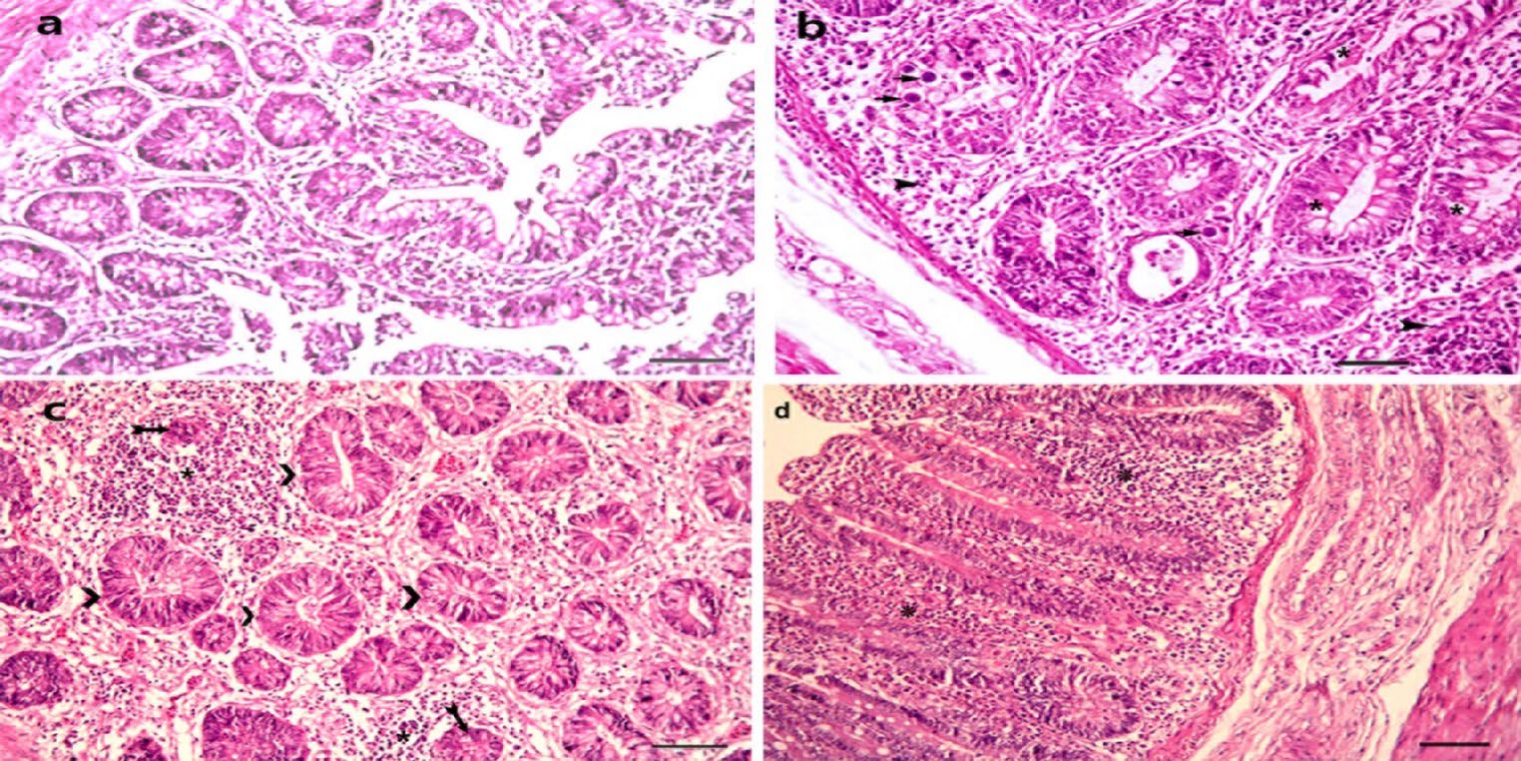
Cecum of chicken treated with Aloe Vera and infected with Eimeria tenella (**G4**) after 7- days from infection (**a**) showing goblet cells hyperplasia in surface and glandular crypt epithelium with very low number of Eimeria stages in the crypt epithelium and inflammatory cells infiltration H& E, bar = 20 µ. (**b**) Cecum from the same group showing goblet cells hyperplasia and degeneration and necrosis of some glandular epithelium (*) few number of Eimeria stages in some glandular epithelium (arrow) with inflammatory cells infiltration (arrow head) H& E, bar = 20 µ. (**c**) Cecum from the same group showing degenerated glandular cells (arrow) surrounded with heavy infiltration of inflammatory cells (*) notice most of the mucosa glands (arrow) H& E, bar = 20 µ. (**d**) Cecum from the same group showing Eimeria stages in some mucosal glandular epithelium H& E, bar = 20 µ

These findings suggest that treatment with Aloe vera gel extract may provide adequate protection against E. tenella infection by reducing the number of Eimeria developmental stages in the tissues and causing less severe histopathological alterations than in untreated birds. The presence of hyperplastic epithelial goblet cells suggests that Aloe vera gel extract may have a protective effect on the intestinal mucosal barrier, which may contribute to its efficacy in preventing coccidiosis in poultry (Fig. 4).

**Fig. 5:**
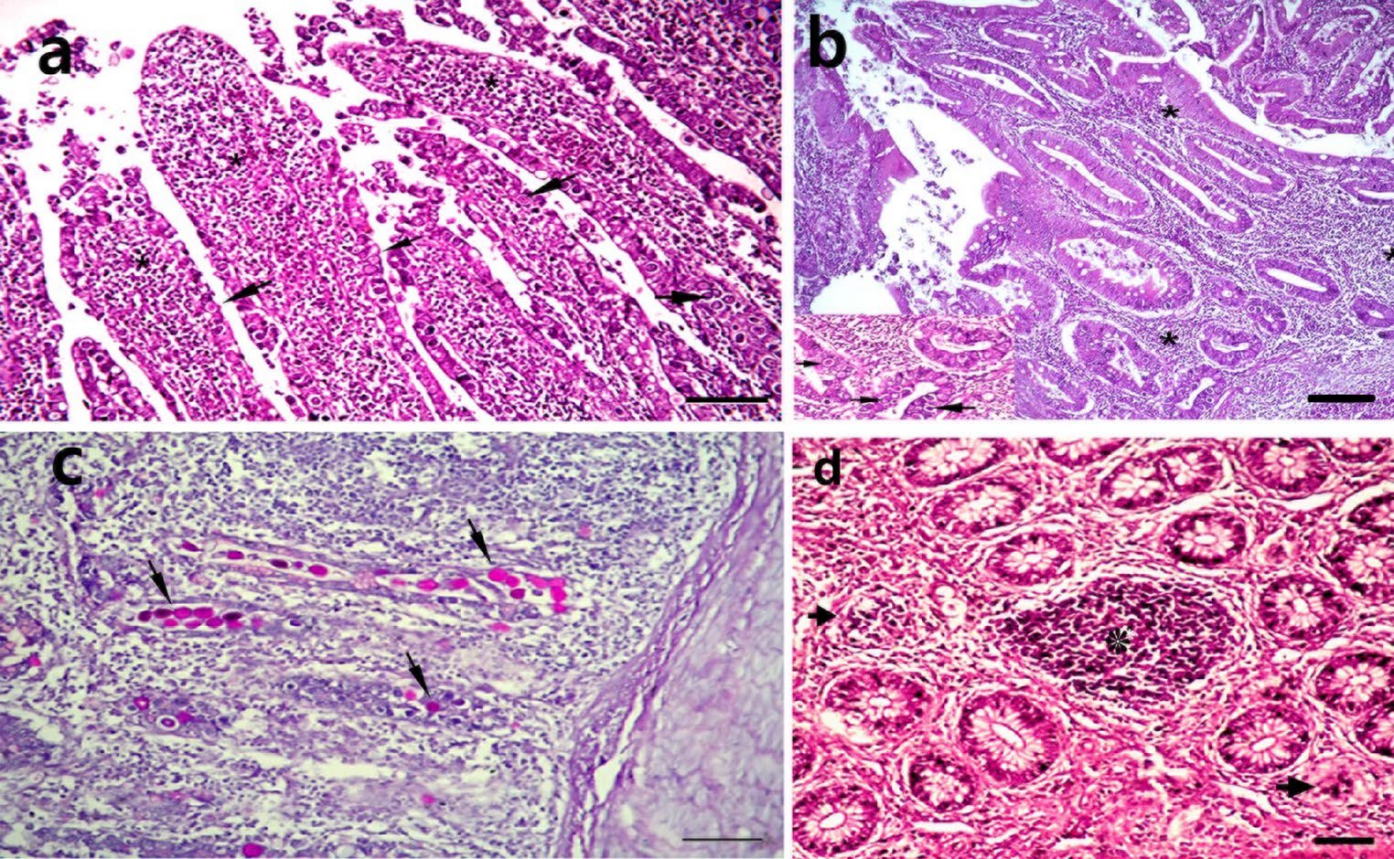
The cecum of vaccinated, challenged chicken and treated with *Aloe vera* (G3), after 7- days from infection (**a**) showing desquamation of epithelial lining in the tips of cecal villi, marked mononuclear cells infiltration (*), and presence of early developmental stages of Eimeria (gametes) (arow) H& E, bar = 20 µ. (**b**) Cecum from the same group showing hyperplasia of mucosal epithelium and sub mucosal glandular epithelium, marked mononuclear and heterophilic cells infiltration (*), and few developmental stages of Eimeria in some epithelium (arrow) H& E, bar = 40 µ. (**c**) PAS positive parasitic stages (arrow) in some crypt, glandular epithelium H& E, bar = 20 µ. (**d**) Goblet cells hyperplasia, with focal leukocytic aggregation (*) in lamina propria and around necrotic mucosa glands (arrow) H& E, bar = 10 µ

The histopathological examination of the cecum of vaccinated, challenged chicken and treated with Aloe vera (G3) revealed desquamation of the epithelial lining in the tips of the caecal villi, goblet cell hyperplasia, and marked inflammatory cell infiltration. Degenerated schizonts with leukocytic inflammatory cells in the lamina epithelial, with a moderate number of Eimeria developmental stages within the villi’s enterocytes, were also demonstrated by PAS stain in the submucosal glandular epithelium. At 15 days post-infection, focal inflammatory cell aggregation and a few numbers of Eimeria oocysts in between submucosal glands were observed, indicating a mild histopathological response compared to the earlier time point. These findings suggest that vaccination before infection with E. tenella and treatment with Aloe vera gel extract may provide some degree of protection against the parasite. This led to a reduction in the number of Eimeria developmental stages in the tissues and a milder histopathological response compared to non-vaccinated and non-treated birds (Fig. 5).

Goblet cell hyperplasia suggests that Aloe vera gel extract may have a protective effect on the intestinal mucosal barrier, which may contribute to its efficacy in controlling coccidiosis in poultry. In general, the results of the histopathological analysis support the use of Aloe vera gel extract as a natural supplement for preventing Eimeria infection in vaccinated and challenged chickens.

**Fig. 6:**
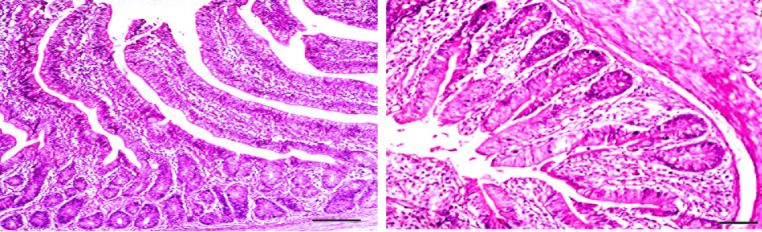
The cecum of non-vaccinated, non-challenged and non- treated chicken (G8), (**a**) showing normal healthy mucosal lining H& E, bar = 40 µ. (**b**) The cecum of non-vaccinated, non-challenged chicken and treated with *Aloe vera* (G)) showing normal healthy mucosal lining similar to healthy non treated control birds H& E, bar = 20 µ

A histopathological examination of the cecum of a control, non-infected chicken and an Aloe vera-treated chicken (G9) revealed a normal, healthy glandular and villus epithelium with normal length and proportions. No histopathological alterations, inflammatory cell infiltrations, or Eimeria developmental stages were observed. These results suggest that treatment with Aloe vera gel extract alone has no negative effects on intestinal tissue and may contribute to the maintenance of a healthy intestinal mucosal barrier in chickens (Fig. 6).

Overall, the histopathological examination results indicate that Aloe vera extract may protect the cecal mucosa and reduce the severity of caecal lesions in broiler chickens caused by E. tenella infection. Additional research is required to confirm these findings and determine the optimal dosage and duration of Aloe vera extract supplementation.

## Discussion

This study assessed the efficacy of an anticoccidial vaccine, and Aloe vera extracts in broiler chickens infected with E. tenella. The results revealed that infected broiler chicks supplemented with Aloe vera had improved growth performance, decreased oocyst shedding, and decreased caecal lesion scores. Combining anticoccidial vaccines with Aloe vera showed promise for addressing drug resistance and residues.

Chickens in the positive control group (G2) infected with E. tenella but not treated exhibited lower body weight gain compared to the other experimental groups, indicating impaired feed intake (FI) and feed conversion ratio (FCR), as reported by Mohammed et al. (2021). Due to its detrimental effects on feed absorption and digestion, avian coccidiosis, which is characterized by watery and bloody diarrhea, is a significant factor contributing to body weight loss and decreased growth performance (Dkhil 2013; Gres et al. 2003). Furthermore, Eimeria infection can lead to varying degrees of parasite-induced necrotic enteritis, which creates an environment favorable for the proliferation of Clostridium perfringens, a highly dangerous pathogen (Wade and Keyburn 2015).

Several studies have investigated the use of natural plants and their extracts to inhibit and suppress the sporulation of E. tenella oocysts (del Cacho et al. 2010; Molan et al. 2009; Yong et al. 2020; Zaman et al. 2012). In the present study, Aloe vera gel extract successfully reduced sporulated oocysts and produced superior results compared to the vaccinated group. The reduction in mean oocysts per gram of feces (OPG) values attained by Aloe vera gel extract was comparable to the best-performing group (G6) treated with amprolium, an effective anticoccidial medication. Compared to the positive control group (G5), G6 demonstrated the most significant (p ≤ 0.05) decrease in mean OPG values. Similar findings have been reported by Mwale et al. (2005) and Narsih and Wignyanto (2012). Aloe vera gel is rich in various biologically active components such as carbohydrates, steroids (Ni and Tizard 2004), minerals, Acemannan, enzymes, Chromones, saccharides, and vitamins C, E, A, B12, B1, and B2 (Lawless & Allen 2000). It also contains proteins such as lectins and lectin-like substances, as well as amino acids like alanine, arginine, aspartic acid, and glutamic acid (Choi and Chung 2003), along with other miscellaneous organic compounds. Additionally, Aloe vera significantly enhances the growth performance of chickens by improving body weight (BW) and body weight gain (BWG) without inducing BW losses. Furthermore, it mitigates the adverse effects of vaccines on body performance, as vaccinated chickens often exhibit lower BW and BWG (Arczewska-Włosek and Świątkiewicz 2014; Lehman et al. 2009).

The positive control group (G5) in our study, consisting of birds infected with E. tenella, exhibited typical clinical signs associated with E. tenella challenge, including depression, weakness, pallor, bloody diarrhea, anorexia, and ruffled feathers. Additionally, there was a significantly elevated caecal lesion score, and the mortality rate reached 24% (Awais et al. 2012; Kim et al. 2013; Yong et al. 2020). Histopathological examination of the caecal lesions in G5 revealed necrosis, sloughing of epithelial cells, congested blood vessels, hemorrhage in the mucosa and submucosal layers, fibrous connective tissue proliferation, and edema. Numerous developmental stages of the parasite, such as oocysts, gametes, zygotes, schizonts, and free merozoites, were observed in large numbers, consistent with previous descriptions (Chanie et al. 2009; Ellakany et al. 2011). Notably, clusters of large schizonts were also present in the cecum, considered second-generation schizonts responsible for extensive tissue damage, bleeding, disruption of caecal glands, and destruction of the mucosa and muscular layers (Ali et al. 2019).

Interestingly, chickens receiving Aloe vera as a prophylactic measure (G4) exhibited reduced caecal lesion scores, lower mortality (12%), decreased oocysts per gram (OPG) values, and mild to moderate diarrhea compared to the positive control group (G5). Histological examination of the cecal tissue in G4 revealed normal cecal villi, mild necrosis and degeneration of the submucosal glands, degenerated oocysts, and a decreased number of Eimeria stages in the mucosa and submucosa. These findings suggest that the growth and development of E. tenella were suppressed or negatively affected by Aloe vera. This finding may be attributed to the antiprotozoal activity of Aloe vera gel, which contains biologically important compounds and exhibits anti-inflammatory, anti-allergic, and antioxidant properties (Hu et al. 2003). Aloe vera also possesses antiseptic properties (Suga and Hirata 1983), antibacterial effects (Alemdar and Agaoglu 2009), as well as antiviral and antifungal properties (Davis 1997), which contribute to healing and protecting infected host tissues from injuries caused by E. tenella and other pathogens.

Aloe vera demonstrated efficacy comparable to or superior to the anticoccidial drug amprolium alone (G6) or in combination with the anticoccidial vaccine Coccivac B (G2) against E. tenella infection. Incorporating Aloe vera gel as a prophylactic treatment in G4 or in combination with oocysts B in G3 resulted in improved caecal histopathological lesions, characterized by the destruction of numerous schizonts, the disappearance of Eimeria stages, and a reduction in the number of Eimeria oocysts.

Similar conclusions were drawn by Dardi (2018), McAllister et al. (1998), and Patterson and Burkholder (2003), who described the synergistic effect of combining a coccidial vaccine with probiotics, prebiotics, and natural products like Yucca schidigera extract. This combination helps preserve the integrity of intestinal villi, enhances weight gain, and improves feed conversion ratio.

A significant difference was observed among the experimental groups after 9 days of infection regarding red blood cell count and hemoglobin concentration. Aloe vera without infection resulted in the highest hemoglobin concentration and red blood cell count, significantly higher than the non-infected non-treated control group. Conversely, the infected non-treated group (G5) exhibited the lowest values. The decrease in red blood cell count is attributed to severe bleeding and tissue damage in the intestinal mucosal surface during the acute stage of infection, as well as the release of large quantities of histamine, which increases capillary and venule permeability, leading to fluid exudation (Padmavathi and Muralidharan 1986).

Group 6 (treated with amprolium) showed the highest hemoglobin concentration and red blood cell count, followed by Aloe vera, with no significant differences observed among the experimentally infected and treated birds. These findings are consistent with those of Haider and AL-Saegh (2018), who reported that hemoglobin concentration and red blood cell count decreased significantly in the untreated, infected group, whereas the addition of Aloe vera juice to drinking water led to a significant increase in Hb and RBC values compared to the control groups.

The improvements observed in hematological and histological parameters can be attributed to the anticoccidial effects of Aloe vera and its content of biologically important compounds, including anti-inflammatory, anti-allergic, and antioxidant activities (Hu et al. 2003), as well as antiseptic properties (Suga and Hirata 1983), antibacterial effects (Alemdar and Agaoglu 2009), and antiviral and antifungal properties. Furthermore, Talmadge et al. (2004) stated that Aloe vera carbohydrates exhibit hematopoietic stimulating activities.

In our study, the infected groups had a higher total leukocyte count, which can be directly attributed to the infection, as infections typically cause an increase in leukocyte count. After nine days of infection, however, more effective treatments led to a reduction in leukocyte count. These results are consistent with those of Melkamu et al. (2018), who reported a significant increase in the total leukocyte count in E. tenella-infected broilers.

Conversely, Aloe vera treatment in the infected group decreased the total leukocyte count compared to the infected non-treated birds. This finding is consistent with the studies by Mahdavi et al. (2012) and Yadav et al. (2017). The decrease in leukocyte count in the infected treated group suggests that the reduction in parasite load (due to the effect of Aloe vera) resulted in the down-regulation of immune system activity, leading to a decrease in the inflammatory response and, subsequently, a decrease in total leukocyte count.

In our study, chickens challenged with E. tenella infection (G5) exhibited a significant up-regulation of IFN-γ, NO, and IL-4 levels nine days post-infection compared to the negative control group (G8). These cytokines are known to be directly involved in the immune response to E. tenella infection, and similar findings have been reported previously (Hong et al. 2006; Tian et al. 2014). The increased production of these cytokines in response to E. tenella infection is attributed to the up-regulation of their transcription levels (Hong et al. 2006; Yun et al. 2000). It is believed that IFN-γ, which is produced by natural killer T cells, stimulates neutrophils and macrophages to migrate from circulation to the site of infection in order to destroy Eimeria sporozoites. Additionally, NO is involved in immunity and resistance against infectious diseases, as it exhibits toxicity towards certain parasites and bacteria (Allen and Fetterer 2000). The significant down-regulation of these cytokine levels in G4 (infected Aloe vera treated) compared to the positive control group (G5) confirms the anti-inflammatory effect of Aloe vera extracts. The increase in serum NO level in G9 (unchallenged Aloe vera treated) compared to the negative control group (G8) further supports the direct role of Aloe vera in elevating NO levels in G4 (challenged and Aloe vera treated), leading to a reduction in inflammation (Allen and Fetterer 2002).

Overall, the anticoccidial effect of Aloe vera in our study was comparable to or even superior to other herbal remedies that have been considered effective for coccidiosis control (Messaï et al. 2014; Yong et al. 2020; Zaman et al. 2012). Aloe vera supplementation significantly reduced fecal oocyst shedding by increasing NO levels and decreasing IL-4 and IFN-γ levels, decreasing inflammation and mitigating the detrimental effects of Eimeria challenge on growth performance, tissue injuries, and lesion scoring. Therefore, Aloe vera is a viable alternative therapy for avian coccidiosis control, as it reduces sporulated oocyst shedding and chicken mortality.

## Conclusions

Aloe vera gel extract appears to be an effective and safe alternative to commercial drugs for controlling Eimeria infection in chickens. Its administration led to a significant increase in weight gain with reductions in oocyst shedding and improvement of histopathological lesion scoring, indicating its potential as an alternative anticoccidial herbal extract of low cost with no adverse side effects in coccidiosis control. In addition, Aloe vera gel extract possesses hematinic effects and other advantageous biological properties, making it a promising natural additive for poultry production.

In addition to its use as a substitute anticoccidial agent, Aloe vera gel extract may provide additional benefits for poultry production. It has been demonstrated, for instance, to enhance nutrient utilization, feed conversion efficiency, and growth performance in broiler chickens. Aloe vera gel extract may have immune-boosting properties that protect chickens from infectious diseases.

Overall, the use of Aloe vera gel extract in poultry production is a promising area of study. However, additional research is required to fully explore its potential benefits and establish the optimal dosage and treatment duration.

### Electronic supplementary material

Below is the link to the electronic supplementary material.

Supplementary Material 1

## Data Availability

All the data are provided in the manuscript.

## Code Availability

N/A.
