# Supplementary material for: Anticoccidial activity of Aloe Vera Leafs’ aqueous extract and vaccination against Eimeria tenella: pathological study in broilers
Source: Vet Res Commun. 2023 Sep 22;48(1):403–16. doi: 10.1007/s11259-023-10222-x (PMC10811142; doi:10.1007/s11259-023-10222-x)

**Supplementary Table 1. Mean body weights (g/bird) of chicks infected with *Eimeria tenella***

**on day 20.**

| **G**  **Age** | **G1**  **(Vaccinated & non- infected)** | **G2**  **(Vaccinated & infected)** | **G3**  **(Vaccinated, infected & *Aloe vera* treated)** | **G4**  **(Infected & treated with Aloe vera)** | **G5**  **(Infected & non- treated)** | **G6**  **(Infected & treated with**  **Amprolium)** | **G7**  **(Treated with amprolium & not infected)** | **G8**  **(Control negative)** | **G9**  **(Treated with Aloe vera& not infected)** |
| --- | --- | --- | --- | --- | --- | --- | --- | --- | --- |
| **Zero day** | 50.9 | 51.4 | 51.1 | 51.1 | 51.3 | 51.2 | 51.1 | 51.4 | 50.9 |
| **1^st^ week** | 271 | 272.9 | 270.9 | 275.2 | 274 | 274 | 272.2 | 274 | 276 |
| **2^nd^ week** | 499 | 497.9 | 495.9 | 510.5 | 505.2 | 504 | 501.5 | 503 | 515 |
| **3^rd^ week** | 1010 | 1008 | 1000 | 1100 | 1015 | 1038 | 1126 | 1117 | 1160 |
| **4^th^ week** | 1690±  19.58^b^ | 1540±  18.71^c^ | 1535±  21.02^c^ | 1580±  21.21^c^ | 1395±  22.55^d^ | 1585±  31.22^c^ | 1812±  14.63^a^ | 1793±  19.30^a^ | 1850±  17.80^a^ |
| **5th week** | 2400±  7.07^c^ | 2240±  15.81^d^ | 2229±  24.27^d^ | 2247±  18.41^d^ | 2015±  13.23^e^ | 2285±  35.24^d^ | 2524±  30.59^b^ | 2515±  44.44^b^ | 2630±  21.21^a^ |

**Supplementary Table 2. Mean body gain (g/bird) of chicks infected with *Eimeria tenella* on day 20.**

| **Grps**  **weeks** | **G5:**  **Infected, non- treated** | **G6 :**  **Infected, treated with amprolium** | **G4:**  **Infected, treated with *Aloe vera*** | **G2:**  **Vaccinated**  **, infected** | **G3:**  **Vaccinated, *Aloe vera* treated, infected** | **G8:**  **Blank control** | **G7:**  **Treated with amprolium**  **, not infected** | **G9:**  **Treated with *Aloe vera*, not infected** | **G1:**  **Vaccinated**  **, non- infected** |
| --- | --- | --- | --- | --- | --- | --- | --- | --- | --- |
| **1^st^ week** | 267.2 | 269.58 | 264.4 | 272.4 | 263.76 | 269.34 | 265.3 | 265.6 | 268.5 |
| **2^nd^ week** | 325.9 | 328.9 | 336.47 | 324 | 328.5 | 327.47 | 325.6 | 332.2 | 330.6 |
| **3^rd^ week** | 708.62 | 720.9 | 789.9 | 795.7 | 781.3 | 798.2 | 805.6 | 786.9 | 797.16 |
| **4^th^ week** | 790 | 897.08 | 792 | 893.7 | 888.1 | 1000.4 | 1008 | 931.5 | 1006.5 |
| **5th week** | 1184.2 | 1253 | 1200 | 1288 | 1263 | 1140 | 1139 | 1209 | 1171 |
| **Total feed intake** | 3275.9 | 3469.5 | 3382 | 3573 | 3524 | 3535 | 3543 | 3525 | 3573 |

**Supplementary Table 3. Feed intake/gm/chick in different experimented groups.**

| **Grps**  **Weeks** | **G1:**  **Vaccinated, non- infected** | **G2:**  **Vaccinated, infected** | **G3:**  **Vaccinated, *Aloe vera* treated, infected** | **G4:**  **Infected, treated with *Aloe vera*** | **G5:**  **Infected**  **, non- treated** | **G6 :**  **Infected, treated with amprolium** | **G7:**  **Treated with amprolium, not infected** | **G8:**  **Blank control** | **G9:**  **Treated with *Aloe vera*,**  **not infected** |
| --- | --- | --- | --- | --- | --- | --- | --- | --- | --- |
| **1^st^**  **Week** | 1.22 | 1.23 | 1.2 | 1.18 | 1.20 | 1.21 | 1.20 | 1.21 | 1.18 |
| **2^nd^**  **Week** | 1.45 | 1.44 | 1.46 | 1.43 | 1.41 | 1.43 | 1.42 | 1.43 | 1.39 |
| **3^rd^**  **Week** | 1.56 | 1.56 | 1.55 | 1.34 | 1.39 | 1.35 | 1.29 | 1.30 | 1.22 |
| **4^th^**  **Week** | 1.48 | 1.68 | 1.66 | 1.65 | 2.08 | 1.64 | 1.47 | 1.48 | 1.35 |
| **5th week** | 1.65 | 1.84 | 1.82 | 1.80 | 1.91 | 1.79 | 1.60 | 1.58 | 1.55 |

**Supplementary Table 4. Feed conversion ratio (FCR) in different experimented groups.**

| **Grps**  **Weeks** | **G1:**  **Vaccinated & non- infected** | **G2:**  **Vaccinated & infected** | **G3:**  **Vaccinated, infected & Aloe vera treated** | **G4:**  **Infected & treated with Aloe**  **vera** | **G5:**  **Infected& non- treated** | **G6 :**  **Infected & treated with**  **amprolium** | **G7:**  **Treated with amproliu m& not**  **infected** | **G8:**  **Blank control** | **G9:**  **Treated with Aloe vera& not**  **infected** |
| --- | --- | --- | --- | --- | --- | --- | --- | --- | --- |
| **1^st^ week** | 220.1 | 221.5 | 219.8 | 224.1 | 222.7 | 222.8 | 221.1 | 222.6 | 225.1 |
| **2^nd^ week** | 228 | 225 | 225 | 235.3 | 231.2 | 230 | 229.3 | 229 | 239 |
| **3^rd^ week** | 511 | 510.1 | 504.1 | 589.5 | 509.8 | 534 | 624.5 | 614 | 645 |
| **4^th^ week** | 680 | 532 | 535 | 480 | 380 | 547 | 686 | 676 | 690 |
| **5th week** | 710 | 700 | 694 | 667 | 620 | 700 | 712 | 722 | 780 |
| **Total body gain** | 2349.1 | 2188.6 | 2177.9 | 2195.9 | 1963.7 | 2233.8 | 2472.9 | 2463.6 | 2579.1 |

**Supplementary Table 5. Hematological parameters in different experimented groups**

| **Groups**  **Parameters** | **G1:**  **Vaccinated, non- infected** | **G2:**  **Vaccinated & infected** | **G3:**  **Vaccinated, infected, *Aloe vera* treated** | **G4:**  **Infected, treated with *Aloe vera*** | **G5:**  **Infected, non- treated** | **G6 :**  **Infected, treated with amprolium** | **G7:**  **Treated with amprolium**  **, not infected** | **G8:**  **Blank control** | **G9:**  **Treated with *Aloe vera*, not infected** |
| --- | --- | --- | --- | --- | --- | --- | --- | --- | --- |
| **HGB**  **(g/dl)** | 10.35  ±  0.14b^cd^ | 9.43\  ±  0.29^d^ | 9.63’  ±  0.23^d^ | 9.58  ±  0.3^d^ | 8.05’  ±  0.38^e^ | 9.85  ±  0.12^cd^ | 10.95  ±  0.079^ab^ | 10.75  ±  0.09^bc^ | 11.80  ±  0.3^a^ |
| **MCH**  **(Pg)** | 40.95  ±  0.44^ab^ | 40.18  ±  0.67^ab^ | 40.63  ±  0.65^ab^ | 41.25  ±  0.52^ab^ | 39.18  ±  3.14^b^ | 42.45  ±  0.87a^b^ | 40.15  ±  0.72^ab^ | 41.78  ±  0.80^ab^ | 46.65  ±  2.97^a^ |
| **MCHC**  **(g/dl)** | 36.53  ±  0.93^a^ | 33.38  ±  0.29^a^ | 35.35  ±  0.68^a^ | 34.63  ±  0.57^a^ | 32.60  ±  0.96^a^ | ^36.75^  ±  0.57^a^ | 34.50  ±  0.65^a^ | 36.05  ±  0.03^a^ | 35.53  ±  2.61^a^ |
| **RBCS (10^6^/ UL)** | 2.95  ±  0.11^b^ | 2.29  ±  0.06^c^ | 2.27  ±  0.04^c^ | 2.34  ±  0.07^c^ | 1.70  ±  0.15^d^ | 2.35  ±  0.13^c^ | 2.92  ±  0.17^b^ | 2.90  ±  0.17^b^ | 3.50  ±  0.19^a^ |

**Supplementary Table 6: Serum concentration of Nitric Oxide (NO), IFN-γ, and Interleukin 4 (IL-4) )at 9^th^ dpi with Eimeria tenella infection on day 20:**

| **Groups** | **G1: Vaccinated, non- infected** | **G2:**  **Vaccinated & infected** | **G3:**  **Vaccinated, infected, *Aloe vera* treated** | **G4:**  **Infected, treated with *Aloe vera*** | **G5: Infected, non-treated** | **G6 : Infected,**  **treated with amprolium** | **G7:**  **Treated with amprolium, not infected** | **G8: Blank control** | **G9: Treated with *Aloe vera*, not infected** |
| --- | --- | --- | --- | --- | --- | --- | --- | --- | --- |
| **NO** | 32.5  ±  3.07^ab^ | 34.5  ±  3.48^ab^ | 33.5  ±  3.43^ab^ | 37.7  ±  4.05^ab^ | 41.5  ±  6.49^a^ | 38  ±  2.38^ab^ | 21.5  ±  2.63^c^ | 23  ±  2.20^bc^ | 26.85  ±  1.12^ab^ |
| **IFN-γ** | 20.28  ±  0.530^d^ | 25.22  ±  0.253^c^ | 27.75  ±  0.380^b^ | 25.15  ±  0.132^c^ | 30.45  ±  0.278^a^ | 18.72  ±  0.266^e^ | 17.25  ±  0.222^f^ | 11.43  ±  0.281^h^ | 14.65  ±  0.422^g^ |
| **IL-4** | 16.95  ±  0.330^a^ | 8.10  ±  .286^d^ | 11.78  ±  0.342b^c^ | 8.55  ±  0.210^d^ | 17.88  ±  0.149^a^ | 8.20  ±  1.903^d^ | 13.68  ±  0.350^b^ | 9.65  ±  0.247^cd^ | 10.2  ±  0.525^cd^ |

Means in the same row with different superscripts are significantly different (p<0.05)

**Supplementary Table 7: Mortality rate following Eimeria tenella infection.**

| **Grps**  **Weeks** | **G1:**  **Vaccinated, non- infected** | **G2:**  **Vaccinated & infected** | **G3:**  **Vaccinated, infected & Aloe vera treated** | **G4:**  **Infected, treated with Aloe vera** | **G5:**  **Infected, non treated** | **G6 :**  **Infected, treated with amprolium** | **G7:**  **Not infected, treated with amprolium** | **G8:**  **Blank, control** | **G9:**  **Treated with *Aloe vera*, not**  **infected** |
| --- | --- | --- | --- | --- | --- | --- | --- | --- | --- |
| **1st week** | **0** | **0** | **0** | **0** | **0** | **0** | **0** | **0** | **0** |
| **2nd week** | **0** | **0** | **0** | **0** | **0** | **0** | **0** | **0** | **0** |
| **3rd week** | **0** | **0** | **0** | **0** | **0** | **0** | **0** | **0** | **0** |
| **4th week** | **0** | **2** | **2** | **2** | **4** | **1** | **0** | **0** | **0** |
| **5th week** | **0** | **1** | **2** | **1** | **2** | **1** | **0** | **0** | **0** |
| **Total mortality** | **0** | **3**  **12%** | **4**  **16%** | **3**  **12%** |  |  |  | **0** |  |

**Supplementary Table 8:** oocyst shedding (OPG) following Eimeria tenella infection**.**

| **Groups**  **Days** | **G1:**  **Vaccinated, non- infected** | **G2:**  **Vaccinated, infected** | **G3:**  **Vaccinated, *Aloe vera* treated, infected** | **G4:**  **Infected,  *Aloe vera* treated** | **G5 (Infected-Untreated)** | **G6 (**  **Infected, amprolium treated )** |
| --- | --- | --- | --- | --- | --- | --- |
| **1 dbi** | 100±17.20^a^ | 80±8.42^a^ | 90±12.91^a^ | 0 | 0 | 0 |
| **6 dpi** | 95±12.41^d^ | 20990±610.7^bc^ | 26700±1329.1^b^ | 19800±1269.5^bc^ | 150000±4112.6^a^ | 14500±451.9^c^ |
| **7 dpi** | 80±5.59^d^ | 44400±2579.7^b^ | 57600±1365.3^b^ | 40000±1521.5^bc^ | 197300±1566.7^a^ | 35800±1885.9^c^ |
| **8 dpi** | 75±2.20^e^ | 24000±1581.1^c^ | 35900±1312.1^b^ | 23800±620.5^c^ | 113000±1416.6^a^ | 10600±540.1^d^ |
| **9 dpi** | 70±2.20^d^ | 11000±449.5^b^ | 14300±501.3^b^ | 10900±513.9^b^ | 78900±1884.6^a^ | 7000±237.2^c^ |
| **10 dpi** | 60±2.38^f^ | 6600±187.1^c^ | 8900±182.6^b^ | 5300±108.01^d^ | 30000±624.01^a^ | 3000±127.9^e^ |

**Supplementary Table 9** Effect of different treatments on fecal score and cecal lesion score of broiler chickens post infection with *Eimeria tenella* at age of 20 days.

| Groups | | | (G2)  Vaccinated & infected | (G3)  Vaccinated, infected, *Aloe vera* treated | (G4)  Infected, treated with *Aloe vera* | (G5)  Infected Untreated | (G6)  Infected, treated with Amprole |
| --- | --- | --- | --- | --- | --- | --- | --- |
| Level of bloody diarrhea* | +  +  ++  +  - | + | + |  |  | + | - |
|  |  | + | + |  |  | ++ | + |
|  |  | ++ | ++ |  |  | +++ | ++ |
|  |  | + | + |  |  | ++ | + |
|  |  | - | - |  |  | + | - |
| Cecal lesion score at day 28 | | | 1.25±0.25^b^ | 1.5±0.28^b^ | 1.25±0.25^b^ | 3.25± 0.25^a^ | 1.0±0.40b |
| Cecal lesion score at day 35 | | | 0.50±0.28^b^ | 0.75±0.25^b^ | 0.50±0.28^b^ | 2.25± 0.47^a^ | 0.25±0.25b |

**Means ± SE in the same row with different superscripts are significantly different (p<0.05).**

**Supplementary Chart 1**


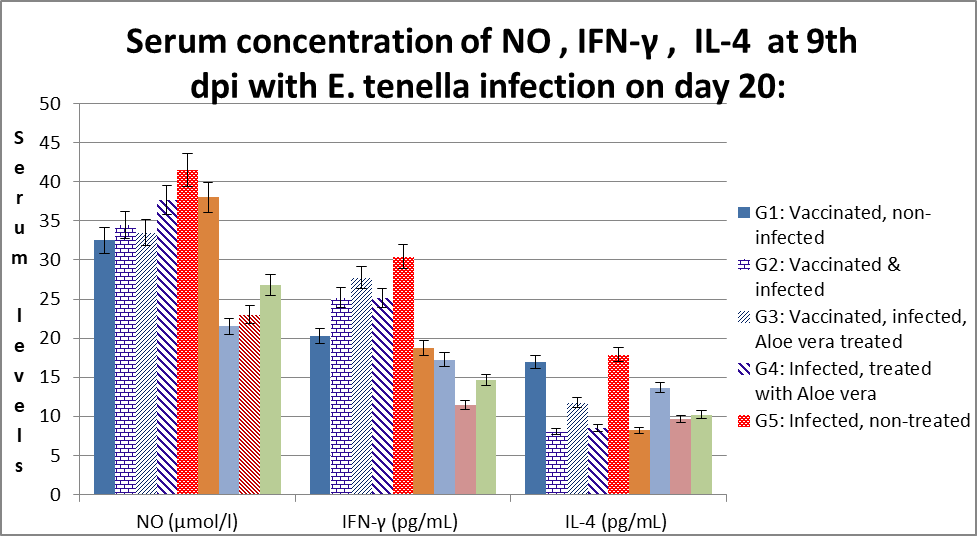


**Supplementary Chart 2**


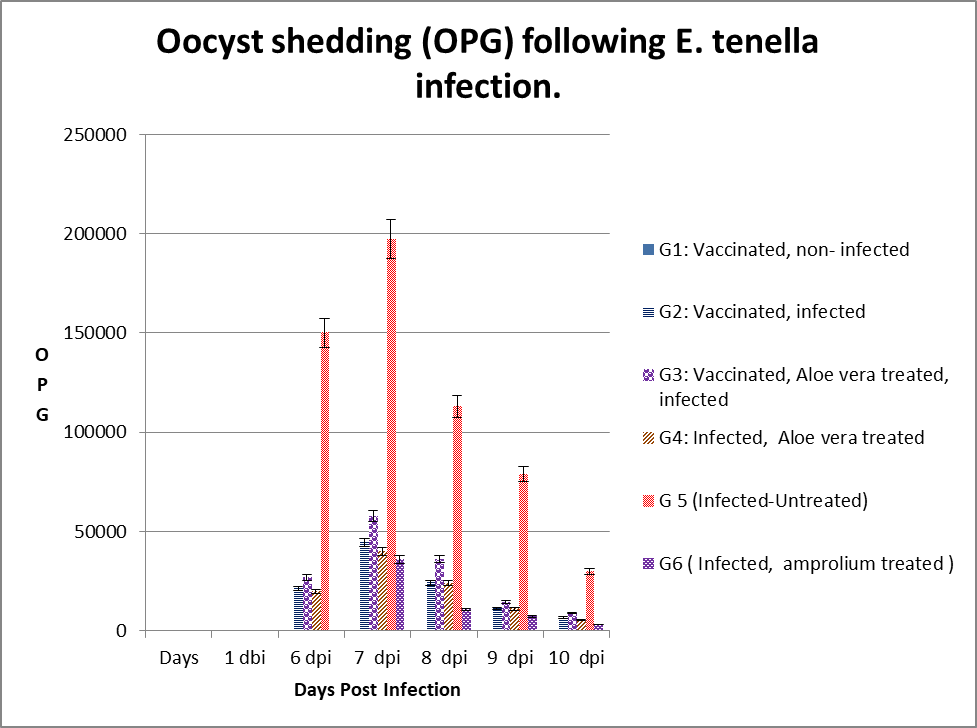

Supplement: Supplementary file 1 — Supplementary Material 1 [file 11259_2023_10222_MOESM1_ESM.doc]
